# Evolution of Entropy in Art Painting Based on the Wavelet Transform

**DOI:** 10.3390/e23070883

**Published:** 2021-07-11

**Authors:** Hongyi Yang, Han Yang

**Affiliations:** 1 School of Mechanical Engeering, Southeast University, NanJing 211189, China; 220194514@seu.edu.cn; 2 School of Aeronautic Science and Engineering, Beihang University, Beijing 100191, China

**Keywords:** art history, paintings, information theory, entropy, wavelet transform, machine learning

## Abstract

Quantitative studies of art and aesthetics are representative of interdisciplinary research. In this work, we conducted a large-scale quantitative study of 36,000 paintings covering both Eastern and Western paintings. The information entropy and wavelet entropy of the images were calculated based on their complexity and energy. Wavelet energy entropy is a feature that can characterize rich information in images, and this is the first study to introduce this feature into aesthetic analysis of art paintings. This study shows that the process of entropy change coincides with the development process of art painting. Further, the experimental results demonstrate an important change in the evolution of art painting, and since the rise of modern art in the twentieth century, the entropy values in painting have started to become diverse. In comparison with Western paintings, Eastern paintings have distinct low entropy characteristics in which the wavelet entropy feature of the images has better results in the machine learning classification task of Eastern and Western paintings (i.e., the F1 score can reach 97%). Our study can be the basis for future quantitative analysis and comparative research in the context of Western and Eastern art aesthetics.

## 1. Introduction

For a long time, aesthetics has been regarded as a philosophical or psychological field. For instance, the Greek philosopher Hippias believed that “beauty is the pleasure produced by sight and hearing”, while the German Enlightenment philosopher Baumgarten defined aesthetics as “the science of sensual perception”. With the rapid development of computer technology, concepts related to computational aesthetics have also been proposed in the context of computer science [1], where researchers hope that computers can learn and simulate human visual and aesthetic habits to quantitatively analyze paintings [2,3], literature [4,5], and music [6,7]. Among them, painting is an important part of art history and an object of study for computational aesthetics in the visual field. Quantitative studies of paintings can not only provide auxiliary information for the appreciation of art [8,9,10] but also enable machines to learn human perceptual behaviors for imitative creation [11,12].

The German aesthete Fechner first introduced experimental psychology to study aesthetics in 1876, and thus the field of experimental aesthetics as a study was created [13]. Inspired by experimental aesthetics, computational aesthetics can be traced back to the American mathematician Birkhoff’s book *Aesthetic Measure*, which was published in 1933 [14]. In this book, Birkhoff argued that beauty in an image is related to its intrinsic order and complexity (i.e., it is proportional to the order within the image and inversely proportional to the complexity of the image). Afterward, Machado et al. extended the definition of aesthetics proposed by Birkhoff [15], while Rigau et al. combined the theory with information theory and analyzed a total of nine paintings by Mondrian, Seurat, and Van Gogh [16]. Since then, fractal analysis has been widely used in the study of fractal art, with the works of the American artist Pollock being used as an object of study [17,18,19,20]. Most subsequent studies have focused on specific painters [21,22] and artworks [23,24] or a particular art movement [25] using a smaller dataset.

With the rapid development of large-scale digital scanning databases and machine vision technology, visual art (and mainly fine art) can be transformed into high-quality digital forms. Thus, it has become possible to extract features from large-scale paintings to carry out further quantitative studies and statistical analysis. The earliest relevant study was conducted by Kim et al. in 2014 on 29,000 paintings from 10 historical periods of Western art. They extracted three color features from the images: the use of a single color, the diversity of colors, and the roughness of brightness. The study found that the color distribution of Western paintings from different historical periods differed significantly and that the roughness index of the images showed an increasing trend over time [26]. Later, this research group carried out a study on the heterogeneity of color distance in modern painting, which pointed out that the difference of this feature is not only caused by regional factors, but also by the transformation of the same artist’s own style [27]. In 2020, they analyzed 14,912 landscapes by 1467 painters from 61 countries based on information theory and network analysis. The results showed that the composition of a landscape painting is different from that of an abstract painting, and the layout of a landscape painting is more regular. In addition, the study found that landscape paintings from different eras or genres varied greatly in the way they were composed, while artists with similar styles often appeared in similar time periods [28]. Sigaki et al. studied the history of art painting through the lens of entropy and complexity. Based on art history spanning 140,000 art paintings over a thousand years, they constructed a complexity–entropy plane which reflected traditional concepts in art history. The study confirmed that artworks from different periods vary significantly in terms of entropy and complexity, and this feature enables the differentiation of different artistic styles and the detection of possible hierarchical organization between them [29].

In this study, the wavelet energy entropy of an image is constructed based on the wavelet transform theory. This feature can describe the richness and complexity of information within an image from the perspective of energy and entropy. In the field of signal processing, the wavelet transform not only has the ability to analyze multi-scale features, but it can also characterize the local features of a signal in both the time and frequency domains, while entropy can describe the uncertainty and instability of a signal [30]. The characteristics of the wavelet entropy can be calculated by applying Shannon entropy processing to the signal after the wavelet transform. The wavelet entropy theory was first proposed by Blanco in 1998 [31]. Since then, related studies have mostly analyzed the transient characteristics of one-dimensional, non-smooth signals, which mainly include fault detection [32,33] and medical diagnosis [34,35]. In recent years, researchers have also applied wavelet entropy theory to image signals and combined it with machine learning techniques for medical diagnosis [36] and facial micro-expression recognition [37]. In this study, 36,000 paintings from art history were studied, belonging to 22 major periods and famous genres in Western art history, as well as Eastern artworks (i.e., mainly ancient Chinese paintings). We extracted the information entropy and wavelet energy entropy for each image based on the entropy and energy of the image. To the best of our knowledge, this is the first study to introduce wavelet energy entropy into the aesthetic analysis of art paintings. Our aim was to see the differences in entropy values between different periods and regions, especially between Eastern and Western paintings. In addition, we want to see if there is a certain regularity in the entropy variation and if that variation coincides with art history-related studies.

## 2. Materials and Methods

### 2.1. Image Information Entropy

Analyzing art paintings based on complexity and entropy has a long history [29,38,39]. In information theory, entropy is a measure of signal uncertainty. Information entropy is a concept used to measure the amount of information, and it can be used to express the value of information from the perspective of its dissemination. It is calculated using the following formula:

(1) $$H\left(X\right)=-\sum_{x}p\left(x\right)logp\left(x\right),$$

where X is a set of random variables and p(x) denotes the probability density of X.

The image information entropy reflects the average information of an image, and it is an important indicator of the richness of the information contained in an image. By calculating the amount of color information in each channel of the image’s color space, the image information entropy can reflect the richness and complexity of the color in that channel. The richer and more complex an image is, the higher the value of the color information.

The information entropy of an image can be calculated from the distribution of pixel values in the image. For a single-color channel, *X* in Equation (1) is the dimension of the color channel, and its probability distribution is as follows:

(2) $$p_{i}=\frac{n_{i}}{N}\left(0\leq i\leq x\right),$$

where $n_{i}$ is the number of pixels in the image with pixel value i, and N is the total number of pixels in the entire image. The one-dimensional information entropy of the image can be determined by combining Equations (1) and (2).

### 2.2. Image Wavelet Energy Entropy

The information entropy of an image characterizes the global average information of different color channels. Furthermore, the two-dimensional entropy of an image can characterize the local information in combination with the spatial distribution of the image. In this study, we combine the image energy and information entropy theories to obtain the two-dimensional wavelet energy entropy of an image by applying the wavelet transform to the image signal. Then, we use Shannon’s entropy theory to obtain the two-dimensional wavelet energy entropy of an image, which reflects the energy distribution of the image in different directions and the complexity of the energy.

**Figure 1:**
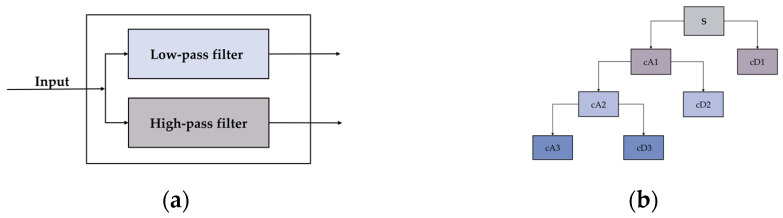
(**a**) Filter structure of the wavelet transform. (**b**) Wavelet decomposition tree of the signal.

The wavelet transform is equivalent to passing the original signal through both low-pass and high-pass filters (Figure 1a). As is shown in Figure 1b, the original signal S could be passed through the low-pass filter to obtain the low-frequency part of the signal cA1, which is the approximate global information of the signal. Through the high-pass filter, the high-frequency part of the signal cD1 is obtained. If the wavelet transform (multi-stage wavelet decomposition) is performed on cA1, obtained by the transform, the low-frequency and high-frequency signals can also be obtained on this basis (cA2, cD2).

**Figure 2:**
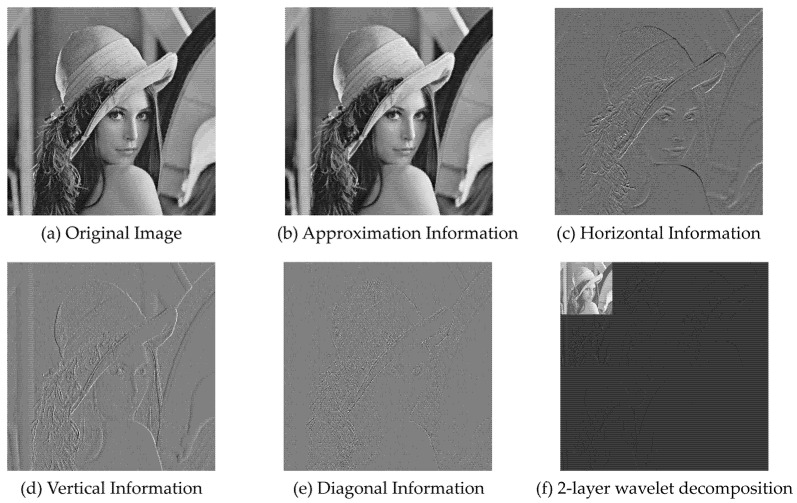
(**a**) The original image, while (**b**–**e**) correspond to the low-frequency information (global approximation), horizontal high-frequency information, vertical high-frequency information, and diagonal high-frequency information after wavelet decomposition, and (**f**) is the image after wavelet decomposition of the low-frequency information in (**b**) and stitching of all coefficients.

If the original signal is considered two-dimensional discrete data $f\left(t_{1},\;t_{2}\right)$ sampled from one image, the image can be decomposed using a two-dimensional discrete wavelet transform. The decomposition process is as follows. First, the horizontal direction of $f\left(t_{1},t_{2}\right)$ and four different frequency sub-bands $\left(LL_{1},HL_{1},LH_{1},HH_{1}\right)$ can be obtained, where $LL_{1}$ is the low-frequency component of $f\left(t_{1},t_{2}\right)$, which is also the global approximate part of the original signal (Figure 2b), $HL_{1}$ is the high-frequency component of $f\left(t_{1},t_{2}\right)$ in the horizontal direction (Figure 2c), $LH_{1}$ is the high-frequency component of $f\left(t_{1},t_{2}\right)$ in the vertica l direction (Figure 2d), and $HH_{1}$ is the high-frequency component of $f\left(t_{1},t_{2}\right)$ in the diagonal direction (Figure 2e).

The wavelet transform can decompose an image into a pyramidal structure containing different frequency sub-bands, and each frequency sub-band after decomposition corresponds to a wavelet transform coefficient $C_{i,j}$. The high-frequency coefficients can reflect the change in the edge details of the image, whereas the low-frequency coefficients can reflect the change in the basic contour of the image. When the wavelet basis functions are a set of orthogonal basis functions, the wavelet transform has an energy conservation property. The wavelet energy of a sub-band is the sum of the squares of the wavelet coefficients presented as follows:

(3) $$E_{i,j}={\left|C_{i,j}\right|}^{2},$$

where *i* is the number of layers of decomposition of the image and *j* is the sub-band of the layer in which it is located (j={LL, HL, LH, HH}). The total energy $E_{Image}$ of the image is the sum of the squares of the wavelet coefficients in each sub-band:

(4) $$E_{Image}=\underset{i}{\sum }E_{i}=\underset{i}{\sum }\underset{j}{\sum }{\left|C_{i,j}\right|}^{2}$$

According to the ratio of the energy of each image sub-band (wavelet relative energy), the wavelet coefficient matrix obtained after the wavelet transform can be processed into a probability distribution sequence. Moreover, the entropy value calculated by this sequence can reflect the sparsity of this coefficient matrix and the energy distribution of the original signal at different scales to obtain the two-dimensional wavelet energy entropy (WE) of the image:

(5) $$WE=-\underset{i,j}{\sum }\frac{E_{i,j}}{E_{Image}}log\frac{E_{i,j}}{E_{Image}}$$

## 3. Results

**Figure 3:**
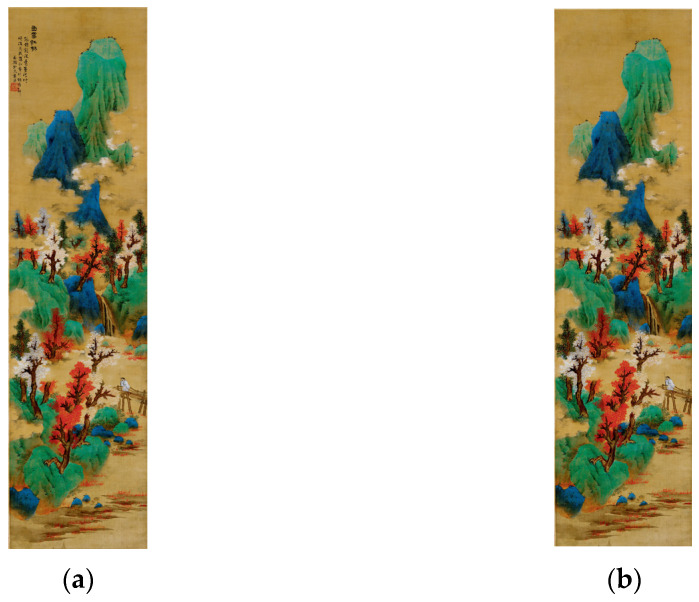
(**a**) Original image. (**b**) Image after removing the poem and seal inside the painting.

The data in this study were mainly obtained from three online databases containing a large number of unwatermarked Eastern and Western art paintings: the Web Gallery of Art [40], Wiki Art [41], and Artbase [42]. Each work selected in the database contained information about the author and the context in which it was created. A sample of 26,000 Western paintings and 8000 Eastern artworks was selected as our dataset. Each of these paintings had a clear indication of its creator and the time of its creation. This would help us in the subsequent analysis and discussion according to the time and genre of each work’s creation. It is also important to note that some of the ancient Chinese paintings were accompanied by poems and seals from the collectors (Figure 3). We manually removed the poems and seals from these paintings to analyze the content of the images.

The Western paintings in the datasets spanned a hundred years, starting with Renaissance paintings and including works from 22 major Western art periods and important schools, including Neoclassicism, Impressionism, and Surrealism. The Eastern paintings in the datasets mainly included paintings from the Tang Dynasty to modern times in China and ukiyo-e works from the Edo period in Japan. Table 1 and Table 2 provide statistics on the number of Western and Eastern painters and their works in the dataset, respectively.

In this study, the information entropy and wavelet energy entropy of images within the six color channels (R, G, B, H, S, and V) of the two most commonly used color spaces, RGB and HSV, were calculated using MATLAB 2021a.

### 3.1. Western Art Painting Information Entropy

The information entropy of an image reflects the richness and complexity of the color in the color channel. This can be calculated according to Equations (1) and (2), where the dimensions of the three color channels R, G, and B are 256 and the channel dimensions of H, S, and V are 180.

**Figure 4:**
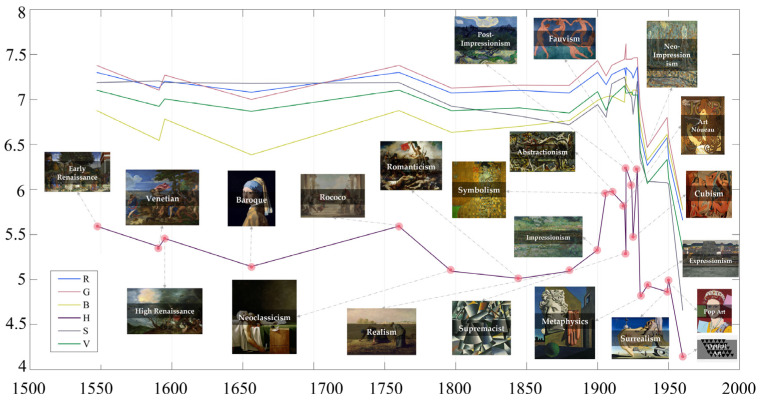
Information entropy evolution curves. (We labeled the entropy change curves in the H channel for different art styles.)

In this study, the mean values of the information entropy of 22 major periods and genres of paintings in the datasets were calculated according to the time span (from the fifteenth century to the middle of the twentieth century), and their trends are shown in Figure 4. It can be seen that the entropy values of art paintings before the twentieth century transformed steadily (from Renaissance to Impressionist paintings). Subsequently, along with the rise of modern art, numerous artistic trends and art schools with avant-garde and pioneering colors emerged. The entropy of these schools differed significantly from that of previous paintings from before the twentieth century. Among them, the abstract expressionism represented by Pollock, Fauvism, which is known for its passion for bright and heavy colors, and Neo-Impressionism (Pointillism), which uses dotted strokes to outline paintings, had higher entropy values. Starting with Supremacist painting, the entropy began to decrease, including Surrealism, Expressionism, and Minimalism.

**Figure 5:**
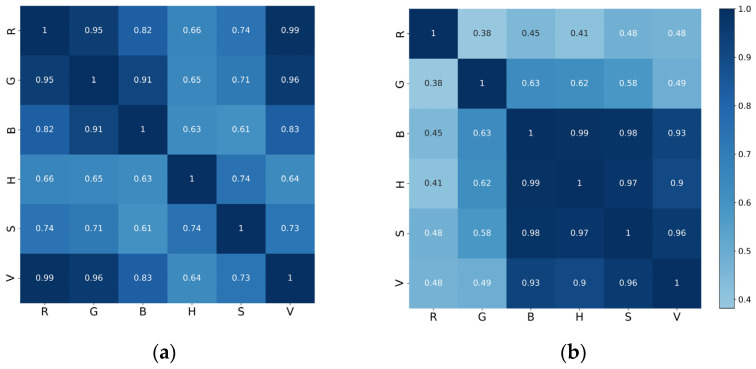
(**a**) The information entropy correlation coefficient matrix between each color channel. (**b**) The wavelet entropy correlation coefficient matrix between each color channel.

In addition, from the above figure, we can see that there was some similarity in the trend of the information entropy values of the six color channels, while the correlation coefficients calculated between the features indicate that all these features were closely correlated (Figure 5a). Among them, the information entropy features between the R, G, and V channels were significantly correlated, and their correlation coefficients were all greater than 0.95.

### 3.2. Western Art Painting Wavelet Energy Entropy

The selection of wavelets is always a key issue in wavelet transform-based research. Different wavelets are used to obtain different wavelet energies and wavelet energy entropies. Although there is no general criterion, the selection of wavelets often depends on the actual situation (e.g., image denoising or image compression). In image processing applications, wavelets are generally required to have symmetry to reduce the phase delay of the processed image so that the edge loss of the processed image is minimized. In this study, a biorthogonal 3.7 wavelet (Bior3.7) was used as the wavelet basis for the calculation.

The image wavelet decomposition is selective by direction and divided into horizontal, vertical, and oblique diagonal directions. This is a characteristic that not only matches the visual properties of the human eye but also enables an understanding of the spatial and frequency structures of the image based on it. The wavelet entropy feature of an image can reflect the energy richness and complexity of the image area and characterize the complexity of texture and color within the image at the same time. A lower entropy value indicates that there is less information or a large amount of similar information (i.e., the same pixel value) in the image, whereas a higher entropy value indicates that the pixels in the image are more different, the texture and color are more complex, and the energy distribution is more disordered.

Usually, the bigger the order of wavelet decomposition, the larger the overall feature vector will be, and the accuracy of the related calculation will be improved. However, for high-resolution image data such as art paintings, multiple orders of wavelet decomposition not only increase the computational effort significantly but also cause the appearance of false textures due to multiple wavelet decomposition. Therefore, four layers of wavelet decomposition were performed for different color channels in each image in this study. Each image was decomposed to obtain 13 different frequency sub-bands, and the two-dimensional energy entropy of each image was calculated according to Equations (3) and (4).

**Figure 6:**
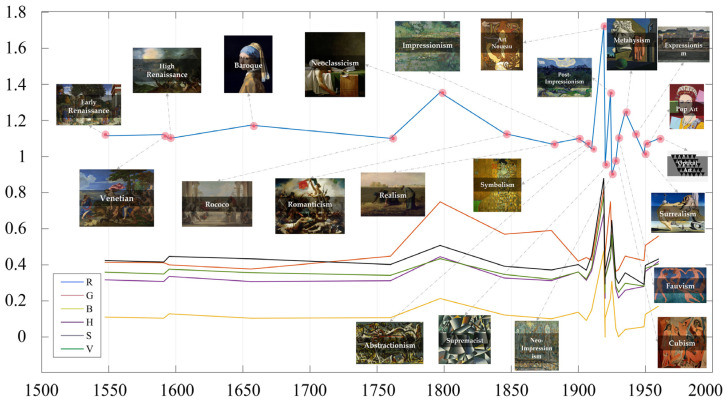
Wavelet energy entropy evolution curves. (We labeled the entropy change curves in the R channel for different art styles.)

As in the previous experiment, we plotted the change curve of the wavelet entropy characteristics of Western art history according to the time span (Figure 6). It can be seen that the wavelet entropy value of the R channel was significantly higher than that of the other color channels, and the wavelet entropy value of the B color channel was the lowest; however, the change trend of each channel was approximately the same. Before the twentieth century, the wavelet entropy of art painting was stable, among which the wavelet entropy of Neoclassicism, which emerged in the eighteenth century, had significantly higher entropy values in the R and G channels than other genres in this period. In addition, since the rise of modernism, the wavelet entropy values of the images began to show a more obvious diversity, in which metaphysical, Neo-Impressionist, and Expressionist paintings had higher entropy values, while Post-Impressionist, Cubist, and Fauvist paintings have significantly lower entropy values. Furthermore, the correlation coefficients between the features are shown in Figure 5, in which the wavelet entropy features of the four color channels (B, H, S, and V) were strongly correlated with each other, while the correlation between the R and G channels and the rest of the channel features was low.

### 3.3. Comparison of Eastern and Western Art Paintings

**Figure 7:**
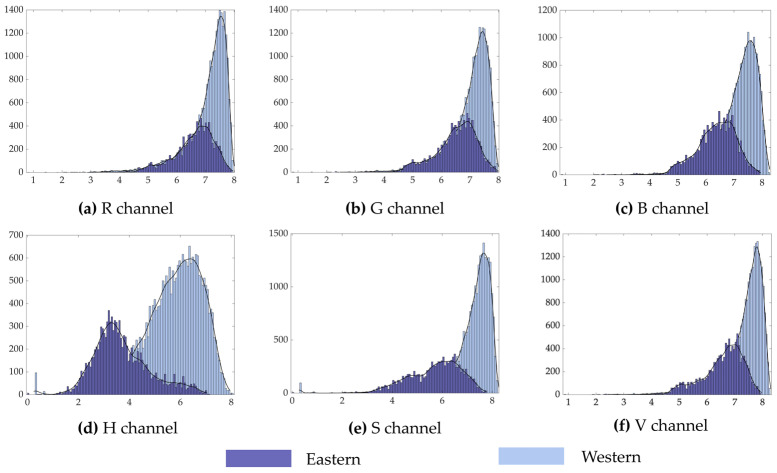
Comparison of the information entropy values of Eastern and Western paintings.

**Figure 8:**
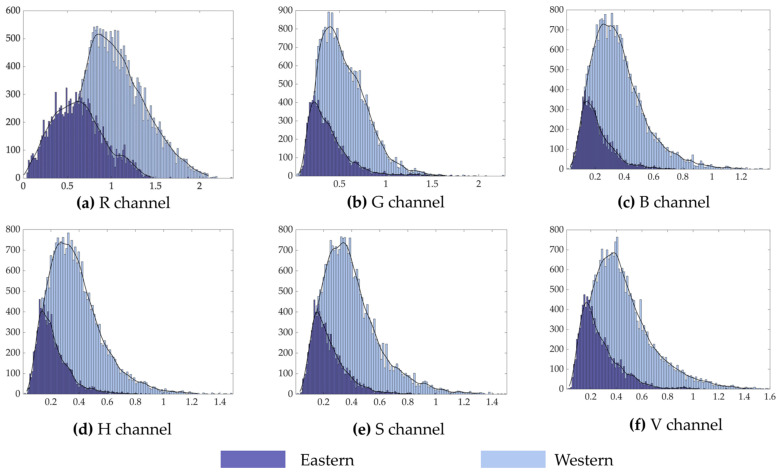
Comparison of the wavelet entropy values of Eastern and Western paintings.

In this study, we compared the entropy values of Eastern and Western art paintings(Figure 7 and Figure 8). First, in comparison with Western paintings, Eastern paintings had lower information entropy and wavelet energy entropy. Second, from the distribution graph of the information entropy values of both cases, the difference in the entropy values of the Eastern and Western paintings in the H channel was lower, and simultaneously, there were obvious differences in the distribution of entropy values compared with the other channels. Meanwhile, the distribution of information entropy values of the Western paintings, in addition to the H channel, was more concentrated, and its values were in the interval of 6.3–8. Third, in terms of the wavelet energy entropy, the distribution of Eastern and Western paintings in the R channel was more different and had obvious differences compared with the distribution of other channels.

To further quantify the two types of entropy information, we conducted classification experiments based on the entropy of images to test whether their information entropy and wavelet entropy could achieve style prediction for Eastern and Western paintings. Three sets of experiments were conducted: classification using information entropy, wavelet entropy, and both types of features. In this study, we used four machine learning classifiers, (support vector machine (SVM) [43], AdaBoost [44], random forest [45], and parsimonious Bayes [46]) to classify art paintings from the East and West, using the 10-fold cross-validation and F1 scores as indicators. Both the random forest and SVM classifiers used a random search method to determine the hyperparameters in the model. The classification results are presented in Table 3.

**Figure 9:**
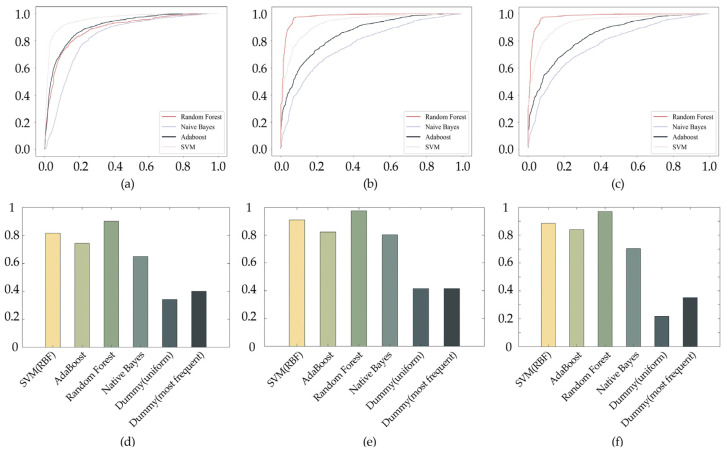
Classification results: (**a**–**c**) ROC curves of three sets of experiments. (**d**–**f**) F1 score comparisons of different classifiers in three sets of experiments.

In comparison with the information entropy feature, the wavelet entropy feature had a better effect on the classification results for the task of classifying East and West paintings. However, when both information entropy and wavelet entropy were used as image features, there were no significantly better classification results. The main reason for this result is that the wavelet entropy feature of the image can characterize both the color and texture complexity of the image, and this feature already contained the information within the information entropy within the image. In addition, after combining the ROC curves with the F1 scores, the SVM classifier had higher classification accuracy than the random forest classifier. All three sets of classification experimental tasks had better classification results than the two random dummy classifiers (Figure 9).

A random forest classifier was used to compare the relative importance of the features in the classification task. The H-channel entropy value in the information entropy-based classification task had higher importance among the six information entropy features. The R-channel and H-channel entropy values in the wavelet entropy-based classification task had a higher degree of importance, whereas in the classification task based on the above two entropy features, there was no significant difference between the importance of the six color channels of the sample, totaling 12 features (the relative importance of each feature after normalization was in the range of 0.08–0.13).

## 4. Discussions

Finding patterns in the evolution of art using quantitative methods is an interesting and meaningful research problem. This paper introduced wavelet energy entropy into the aesthetic analysis of art paintings based on entropy and complexity. The study traced the evolution of complexity in Western art paintings and compared it with Eastern paintings based on a total of 36,000 artworks.

First, we considered the paintings of Western art history spanning a century as the main research object and combined Shannon’s entropy theory with image wavelet decomposition techniques to calculate the information entropy and wavelet energy entropy for the six color channels of each image. The research results show that there were certain similarities and regularities in the changes of these two entropies. One of the remarkable patterns was that the entropy changes in Western paintings were more stable before the twentieth century, but with the rise of modern art, the entropy values in the paintings also showed diversity, and the magnitudes of the entropy values were significantly different from those of previous paintings. We speculate that this is mainly due to the fact that most modern art schools, including Expressionism, Cubism, and Surrealism, no longer depict concrete images or objects, and the images often do not contain objects that are familiar to us visually. In addition, the painters of this period also began to pay attention to the spiritual world and inner experience of human beings; thus, more subjective thoughts of the artists were incorporated into the pictures, and the use of colors and compositions no longer abided by traditional theories and techniques, resulting in the pictures being wilder and more disorderly.

Second, we conducted a comparison between Eastern and Western paintings based on Shannon’s entropy and wavelet entropy. The experimental results show that the Eastern paintings had obvious low entropy characteristics, which indicates that the Western paintings had higher richness and complexity in color and texture. At the same time, the information entropy characteristics of the Eastern paintings were more dispersed. We speculate that there are two main reasons for this phenomenon. The first reason is that Eastern painting spans over a thousand years from the Tang Dynasty to modern times, and the painting styles vary greatly from one period to the next. Oriental painting, mainly Chinese painting, contains more of the painter’s own emotions and understanding of the world and promotes “putting emotion in the scene”. In terms of the painting’s expression, it does not pay attention to the light, darkness, and authenticity of color and considers lines as the main body while promoting “ink instead of color”. In contrast, Western painting is based on rigorous science (e.g., using geometric relationships, optical knowledge, anatomy, and other scientific principles to express things and people precisely). Therefore, these differences also cause Eastern paintings, especially Chinese ink paintings, to be less complex than Western oil paintings in terms of composition and color and appear more balanced and simpler visually.

Third, we conducted several machine learning classification experiments on Eastern and Western paintings based on the information entropy and wavelet entropy features of the images. The results show that wavelet entropy, which can simultaneously characterize more information in the images, had a more accurate classification effect. The SVM and random forest classifiers performed better in all three sets of classification tasks, and the relative importance of different color channels in the classification task was also different. We believe that the wavelet entropy feature can quantify the image in the frequency domain, and the size of the value contains rich information about the color, texture, complexity, and energy of the image. We believe that this feature can be used in combination with other image features to achieve better classification and recognition of different categories of images under abstract concepts.

Finally, our study has some limitations. The ancient Chinese paintings and Japanese Edo period ukiyo-e artworks involved in the study were only used for comparison with Western art paintings, and our main object of study was still the paintings of European artists since the Renaissance. In contrast to Western European art history, Eastern art history spans a much longer period of time, with the earliest ancient Chinese paintings dating back to the Warring States period and Japanese ukiyo-e artworks painted and produced in various ways in different periods. Our study can be used as a starting point for future research to focus more on Eastern art, build a larger dataset, and conduct quantitative analysis and research in the context of Eastern art aesthetics. In addition, with the rapid development of digital media, computers have become a new tool for painting, resulting in new forms of art such as computer art and pixel art. At the same time, the expression of art and the public’s understanding of art have also changed dramatically; therefore, conducting quantitative research on art in this new era will be an interesting topic to explore.

## Figures and Tables

**Table 1:** Western painting statistics.

Year	Style	Painters	Paintings
−1600	Early Renaissance	9	757
	Venetian	5	365
	High Renaissance	15	1693
1601–1700	Baroque	14	1400
1701–1800	Rococo	10	943
	Neoclassicism	12	719
1801–1900	Romanticism	10	1258
	Realism	8	1044
	Impressionism	22	2541
	Symbolism	8	669
	Metaphysics	5	302
	Art Nouveau	3	501
	Supremacist	5	259
	Post-Impressionism	12	1825
	Neo-Impressionism	6	358
	Cubism	10	2360
	Fauvism	9	644
	Abstractionism	12	424
	Expressionism	6	513
	Surrealism	9	457
	Pop Art	11	785
	Optical Art	3	254

**Table 2:** Eastern painting statistics.

Nation	Period	Painters	Paintings
China	Tang Dynasty	23	253
	Song Dynasty	53	744
	Yuan Dynasty	44	590
	Ming Dynasty	64	1050
	Qing Dynasty	87	1540
	Modern Times	70	1369
Japan	Edo Period	49	2670

**Table 3:** Classification results.

	SVM (RBF)	AdaBoost	Random Forest	Naive Bayes
Entropy	0.816	0.743	0.731	0.639
Wavelet Entropy	0.885	0.810	0.970	0.702
Entropy and Wavelet Entropy	0.904	0.842	0.976	0.684

## Data Availability

The data used in this paper are from the following 3 publicly available datasets: the Web Gallery of Art (https://www.wikiart.org), Wiki Art (https://www.artbase.cn/), and Artbase (https://www.artbase.cn/).

## References

[1] Hoenig F. Defining computational aesthetics. Proceedings of the 1st Eurographics Conference on Computational Aesthetics in Graphics, Visualization and Imaging.

[2] Manovich L. (2015). Data science and digital art history. Int. J. Digit. Art Hist..

[3] Montagner C., Linhares J.M.M., Vilarigues M., Nascimento S.M.C. (2016). Statistics of colors in paintings and natural scenes. J. Opt. Soc. Am. A.

[4] Reagan A.J., Mitchell L., Kiley D., Danforth C.M., Dodds P.S. (2016). The emotional arcs of stories are dominated by six basic shapes. Eur. Phys. J. Data Sci..

[5] Reagan A.J., Danforth C.M., Tivnan B., Williams J.R., Dodds P.S. (2017). Sentiment analysis methods for understanding large-scale texts: A case for using continuum-scored words and word shift graphs. Eur. Phys. J. Data Sci..

[6] Park D., Bae A., Schich M., Park J. (2015). Topology and evolution of the network of western classical music composers. EPJ Data Sci..

[7] Corrêa D.C., Saito J.H., da F Costa L. (2010). Musical genres: Beating to the rhythms of different drums. New J. Phys..

[8] Falomir Z., Museros L., Sanz I., Gonzalez-Abril L. (2018). Categorizing paintings in art styles based on qualitative color descriptors, quantitative global features and machine learning (QArt-Learn). Expert Syst. Appl..

[9] Zujovic J., Gandy L., Friedman S., Pardo B., Pappas T.N. Classifying paintings by artistic genre: An analysis of features & classifiers. Proceedings of the 2009 IEEE International Workshop on Multimedia Signal Processing.

[10] Johnson C.R., Hendriks E., Berezhnoy I.J., Brevdo E., Hughes S.M., Daubechies I., Li J., Postma E., Wang J.Z. (2008). Image processing for artist identification. IEEE Signal Process. Mag..

[11] Mazzone M., Elgammal A. (2019). Art, Creativity, and the Potential of Artificial Intelligence. Arts.

[12] Lisi E., Malekzadeh M., Haddadi H., Lau F.D.-H., Flaxman S. (2020). Modeling and Forecasting Art Movements with CGANs. R. Soc. Open Sci..

[13] Fechner G.T. (1876). Vorschule der Aesthetik.

[14] Birkhoff G.D. (1933). Aesthetic Measure.

[15] Machado P., Cardoso A. (1998). Computing aesthetics. Advances in Artificial Intelligence. SBIA 1998.

[16] Rigau J., Feixas M., Sbert M. (2008). Informational aesthetics measures. IEEE Comput. Graph. Appl..

[17] Taylor R.P., Micolich A.P., Jonas D. (1999). Fractal analysis of Pollock’s drip paintings. Nature.

[18] Jones-Smith K., Mathur H. (2006). Fractal analysis: Revisiting Pollock’s drip paintings. Nature.

[19] Taylor R.P., Guzman R., Martin T.P., Hall G.D.R., Micolich A.P., Jonas D., Scannell B.C., Fairbanks M.S., Marlow C.A. (2007). Authenticating Pollock paintings using fractal geometry. Pattern Recognit. Lett..

[20] Shamir L. (2012). Computer analysis reveals similarities between the artistic styles of Van Gogh and Pollock. Leonardo.

[21] Hughes J.M., Graham D.J., Rockmore D.N. (2010). Quantification of artistic style through sparse coding analysis in the drawings of Pieter Bruegel the Elder. Proc. Natl. Acad. Sci. USA.

[22] Berezhnoy J.I., Postma E., van den Herik J. (2007). Computer analysis of van Gogh’s complementary colours. Pattern Recognit. Lett..

[23] Pedram P., Jafari G.R. (2008). Mona Lisa, the stochastic view and fractality in color space. Int. Mod. Phys. C.

[24] James B., Kriel N. (2019). Is the Starry Night Turbulent?. arXiv.

[25] Elsa M., Zenit R. (2017). Topological invariants can be used to quantify complexity in abstract paintings. Knowl.-Based Syst..

[26] Kim D., Son S.-W., Jeong H. (2014). Large-scale quantitative analysis of painting arts. Sci. Rep..

[27] Lee B., Kim D., Sun S., Jeong H., Park J. (2018). Heterogeneity in chromatic distance in images and characterization of massive painting dataset. PLoS ONE.

[28] Lee B., Seo M.K., Kim D., Shin I.S., Schich M., Jeong H., Han S.K. (2020). Dissecting landscape art history with information theory. Proc. Natl. Acad. Sci. USA.

[29] Sigaki H.Y.D., Perc M., Ribeiro H.V. (2018). History of art paintings through the lens of entropy and complexity. Proc. Natl. Acad. Sci. USA.

[30] Shannon C.E. (1948). A Mathematical Theory of Communication. Bell Syst. Tech..

[31] Blanco S., Figliosa A., Quian Quiroga R., Rosso O.A., Serrano E. (1998). Time-frequency analysis of electro encephalogram series (III): Information transfer function and wavelets packets. Phys. Rev. E.

[32] Zonkoly A.M., Desouki H. (2011). Wavelet entropy based algorithm for fault detection and classification in FACTS compensated transmission line. Int. J. Electr. Power Energy Syst..

[33] Zhang Y.N., Wei W., Wu L. (2010). Motor mechanical fault diagnosis based on wavelet packet, Shannon entropy, SVM and GA. Electr. Power Autom. Equip..

[34] Hou H., Gui Z.G. (2010). Denoising processing of ECG signal based on wavelet entropy. Chin. J. Biomed. Eng..

[35] Hasan A., Joseph S.P., Wendy C.Z. (2003). Wavelet entropy for subband segmentation of EEG during injury and recovery. Ann. Biomech. Eng..

[36] Kouamou Ntonfo G.M., Ferrari G., Raheli R. (2012). Low-Complexity Image Processing for Real-Time Detection of Neonatal Clonic Seizures. IEEE Trans. Inf. Technol. Biomed..

[37] Wang S.H., Preetha Phillips P., Dongc Z.C., Zhang Y.D. (2018). Intelligent facial emotion recognition based on stationary wavelet entropy and Jaya algorithm. Neurocomputing.

[38] Kosheleva M., Kreinovich V., Yam Y. (1998). Towards the use of aesthetics in decision making: Kolmogorov complexity formalizes birkhoff’s idea. Bull. EATCS.

[39] Boon J.P., Casti J., Taylor R.P. (2011). Artistic forms and complexity. Nonlinear Dyn. Psychol. Life Sci..

[40] WikiArt Visual Art Encyclopedia. https://www.wikiart.org.

[41] Web Gallery of Art. https://www.wga.hu/.

[42] Artbase. https://www.artbase.cn/.

[43] Cortes C., Vapnik V. (1995). Support-vector networks. Mach. Learn..

[44] Freund Y., Schapire R.E. (1997). A Decision-Theoretic Generalization of On-Line Learning and an Application to Boosting. J. Comput. Syst. Sci..

[45] Beriman L. (2001). Random forests. Mach. Learn..

[46] Mitchell T.M. (2005). Chapter 3: Generative and discriminative classifiers: Naïve Bayes and logistic regression. Machine Learning.

